# Supplementary material for: Self-Supervised Learning Improves Accuracy and Data Efficiency for IMU-Based Ground Reaction Force Estimation
Source: bioRxiv. 2024 Jan 25:2023.10.25.564057. Preprint. [Version 2] doi: 10.1101/2023.10.25.564057 (PMC10849467; doi:10.1101/2023.10.25.564057)
Supplement: Supplement 1 [file media-1.pdf]

Supplementary Table I RMSE between the Gold-Standard and Estimated GRF for SSL Pre-trained Model during Stance, Swing Phases of Walking and Landing, Flight Phases of Drop Landing.

		RMSE (*N/kg*)		
Dataset	Phase	mlGRF[a]	apGRF[a]	vGRF[a]
Overground Walking	Stance	0.04±0.01	0.03±0.01	0.13±0.03
	Swing	0.01±0.01	0.01±0.00	0.05±0.02
Treadmill Walking	Stance	0.02±0.00	0.03±0.01	0.12±0.02
	Swing	0.01±0.00	0.01±0.00	0.06±0.01
Drop Landing	Landing	0.04±0.01	0.05±0.01	0.19±0.05
	Flight	0.01±0.00	0.01±0.00	0.05±0.02

[a] ml: medial-lateral; ap: anterior-posterior; v: vertical
